# Construction and Validation of a m7G-Related Gene-Based Prognostic Model for Gastric Cancer

**DOI:** 10.3389/fonc.2022.861412

**Published:** 2022-06-30

**Authors:** Xin-yu Li, Shou-lian Wang, De-hu Chen, Hui Liu, Jian-Xiong You, Li-xin Su, Xi-tao Yang

**Affiliations:** ^1^ Department of Interventional Therapy, Shanghai Ninth People's Hospital, Shanghai Jiao Tong University School of Medicine, Shanghai, China; ^2^ Department of Neurosurgery, Shanghai Ninth People’s Hospital, Shanghai JiaoTong University School of Medicine, Shanghai, China; ^3^ Department of General Surgery, Shanghai Ninth People’s Hospital, Shanghai Jiao Tong University School of Medicine, Shanghai, China; ^4^ Department of Gastrointestinal Surgery, Hospital Affiliated 5 to Nantong University (Taizhou People's Hospital), Taizhou, China; ^5^ Department of Clinical Medicine, Shanghai Jiao Tong University Affiliated Sixth People’s Hospital, Shanghai, China

**Keywords:** gastric cancer, N7-methyladenosine, overall survival, prognostic model, bioinformatics

## Abstract

**Background:**

Gastric cancer (GC) is one of the most common malignant tumors of the digestive system. Chinese cases of GC account for about 40% of the global rate, with approximately 1.66 million people succumbing to the disease each year. Despite the progress made in the treatment of GC, most patients are diagnosed at an advanced stage due to the lack of obvious clinical symptoms in the early stages of GC, and their prognosis is still very poor. The m7G modification is one of the most common forms of base modification in post-transcriptional regulation, and it is widely distributed in the 5′ cap region of tRNA, rRNA, and eukaryotic mRNA.

**Methods:**

RNA sequencing data of GC were downloaded from The Cancer Genome Atlas. The differentially expressed m7G-related genes in normal and tumour tissues were determined, and the expression and prognostic value of m7G-related genes were systematically analysed. We then built models using the selected m7G-related genes with the help of machine learning methods.The model was then validated for prognostic value by combining the receiver operating characteristic curve (ROC) and forest plots. The model was then validated on an external dataset. Finally, quantitative real-time PCR (qPCR) was performed to detect gene expression levels in clinical gastric cancer and paraneoplastic tissue.

**Results:**

The model is able to determine the prognosis of GC samples quantitatively and accurately. The ROC analysis of model has an AUC of 0.761 and 0.714 for the 3-year overall survival (OS) in the training and validation sets, respectively. We determined a correlation between risk scores and immune cell infiltration and concluded that immune cell infiltration affects the prognosis of GC patients. *NUDT10, METTL1, NUDT4, GEMIN5, EIF4E1B*, and *DCPS* were identified as prognostic hub genes and potential therapeutic agents were identified based on these genes.

**Conclusion:**

The m7G-related gene-based prognostic model showed good prognostic discrimination. Understanding how m7G modification affect the infiltration of the tumor microenvironment (TME) cells will enable us to better understand the TME’s anti-tumor immune response, and hopefully guide more effective immunotherapy methods.

## Introduction

Gastric cancer (GC) remains the second leading cause of cancer-related mortality worldwide (1). Patients with advanced GC often experience tumour invasion and metastasis, resulting in a significantly shorter average survival time (2). Despite considerable advances in the field of GC treatment in recent years, our knowledge of GC pathogenesis and progression mechanism remains limited, and the prognosis of GC patients remains relatively poor (3). Therefore, it is crucial to better understand the mechanisms of GC invasion and metastasis while identifying additional diagnostic and prognostic biomarkers associated with GC. The traditional statistical methods are only applicable to the analysis of data that have numerical characteristics and are consistent with statistical patterns (4). The main purpose of machine learning is to discover hidden patterns in the data based on feature information from multidimensional data, mining the connections between features (5). Based on a large amount of data and based on machine learning methods to analyse potential associations between patient indicators, to uncover risk factors for the cure, to further develop a survival prediction model for the disease and to make clear diagnostic recommendations to patients (6). Previous articles on machine learning and GC have demonstrated the role of machine learning in the diagnosis of GC (7, 8).

M7G RNA methylation (N7 methyladenosine, m7G) is a modification in which a methyl group is added to the seventh position N of RNA guanine (G) under the action of methyltransferase (9). M7G modification is one of the most common forms of base modification in post-transcriptional regulation. It is widely distributed among tRNA, rRNA, and the 5′ cap of eukaryotic mRNA, and plays an important role in ‘RNA processing, metabolism, stability, and nucleation, as well as translation (9–11). Previous studies have found that m7G tRNA modification enhances oncogenic mRNA translation and promotes the progression of intrahepatic cholangiocarcinoma (9). However, few studies have verified whether m7G-related genes play a key role in GC. This study attempts to explore the potential functions of m7G and the molecular mechanisms underlying GC infiltration by constructing a prognostic model of aberrantly expressed m7G-related genes in GC samples and normal tissues.

## Material and Methods

### Data Acquisition

From previous systematic reviews and the Molecular Signatures Database, a total of 29 m7G-related genes were extracted (11–14). RNA sequencing datasets of 32 normal gastric tissues and 375 gastric cancer specimens were downloaded from The Cancer Genome Atlas (https://portal.gdc.cancer.gov/), together with the corresponding clinical data. All raw data were preprocessed with the limma R package and screened for differentially expressed genes (DEGs) associated with m7G. A Wilcox test was performed using |logFC(foldchange)| ≥ 1 and false discovery rate (FDR) < 0.05 as the cutoff criteria. The training group comprised data from the TCGA cohort, and the validation group comprised gene expression data and corresponding post-operative data collected from January 2015 to December 2020 in 70 human GC patients admitted at Taizhou People’s Hospital. All participants signed written informed consent forms prior to their participation. The study protocol was approved by the hospital ethics committee. Clinical information including age, gender, and TNM stage was collected (**Tables 1**, **2**).

**Table 1:** Clinical characteristics of the GC patients used in the derivation cohort.

Characteristic	levels	Overall
n		375
T stage, n (%)	T1	19 (5.2%)
	T2	80 (21.8%)
	T3	168 (45.8%)
	T4	100 (27.2%)
N stage, n (%)	N0	111 (31.1%)
	N1	97 (27.2%)
	N2	75 (21%)
	N3	74 (20.7%)
M stage, n (%)	M0	330 (93%)
	M1	25 (7%)
Gender, n (%)	Female	134 (35.7%)
	Male	241 (64.3%)
Age, n (%)	<=65	164 (44.2%)
	>65	207 (55.8%)
Age, median (IQR)		67 (58, 73)

**Table 2:** Clinical characteristics of the GC patients used in the validation cohort.

Characteristic	levels	Overall
n		70
T stage, n (%)	T1	10 (14%)
	T2	15 (38%)
	T3	25 (35%)
	T4	20 (13%)
N stage, n (%)	N0	30 (42%)
	N1	10 (14%)
	N2	17 (24%)
	N3	13 (20%)
M stage, n (%)	M0	56 (79%)
	M1	14 (20%)
Gender, n (%)	Female	35 (50%)
	Male	35 (50%)
Age, n (%)	<=65	30 (42%)
	>65	40 (58%)
Age, median (IQR)		62 (56, 75)

### Development and Validation of the m7G-Related Genes Prognostic Model

R software (R Foundation for Statistical Computing, Vienna, Austria) with the Survival package was used to conduct univariate and multivariate Cox analyses. The optimal prediction model was determined as per the Akaike Information Criterion (AIC). Based on the results of the univariate Cox regression analysis, we used a support vector machine (SVM) approach to further screen for prognostic genes with the help of the R package e1071(http://cran.r-project.org/package=e1071) (15). The risk score for each patient was accorded based on the expression level of m7G-related genes and the regression coefficient β of the weighted linear combination in the multifactorial analysis, calculated as: risk score = βgene1 × expr(gene1) + βgene2 × expr(gene2) +… + βgeneN × expr(geneN). Based on the median risk score, GC patients were divided into low-risk and high-risk groups. The Kaplan-Meier (K-M) method was used to clarify the relationship between risk values and survival time. Furthermore, ROC curves were drawn to assess the predictive effect of the model; and the risk and survival status were plotted for high- and low-risk groups. In addition, principal component analysis (PCA) was performed using the “prcomp” package to visualise the grouping of data and the distribution of different groups. Based on the above risk score formula, risk scores were calculated for patients in the validation group, who were subsequently divided into high- and low-risk groups and analysed using the K-M method.

### Cox Regression Analysis of Prognostic Factors for Gastric Cancer

Risk scores were combined with clinical information including the survival time, survival status, sex, age, tumour grade, pathological grade, TNM stage, and risk score. Clinical factors affecting GC prognosis were initially screened using univariate Cox regression analysis, and factors significantly associated with GC prognosis were subsequently subjected to multivariate Cox regression analysis.

### Enrichment Analysis of DEGs and Drug Sensitivity Analysis

The biological functions of these differentially expressed m7G-genes were examined integrally by Gene Ontology term enrichment analysis (GO) and the Kyoto Encyclopedia of Genes and Genomes (KEGG) analysis (16). All enrichment analyses were done using the org.Hs.eg.db, DOSE, clusterProfiler, and enrichplot packages. P<0.05 while FDR<0.05 was considered a statistically significant difference. The Drug Signatures Database (DSigDB) currently holds 17,389 unique compounds and 19,531 drug target genes, and is freely available at http://tanlab.ucdenver.edu/DSigDB (17). We used the DSigDB database to identify potential drugs that significantly interacted with genes (18). P<0.05 was considered a statistically significant difference.

### Immune Cell Infiltration

Infiltration of immune cells into the TME is known to influence tumour progression (19). A systematic analysis of the relationship between tumour immune infiltration and hub genes can throw light on the mechanism of tumour immune escape and facilitate the development of new biomarkers and therapeutic strategies. Thus, we checked for correlation between the expression of hub genes and immune infiltration in GC. By single-sample gene set enrichment analysis (ssGSEA), the infiltration of 28 immune cells was analysed, with the ssGSEA score set as the standard (20).

### Quantitative Real-Time PCR

The cancerous tissues and normal tissues adjacent to the tumour were surgically excised, added to RNAlater protective solution, and frozen at -80°C for further examination. The total RNA was extracted by the TRIzol method. After centrifugation, 1 μg of total RNA was extracted from the supernatant, and cDNA was synthesised using a cDNA reverse transcription kit. The cDNA was used as the template and the *GAPDH* gene as the internal reference for real-time PCR. A two-step standard PCR amplification procedure was used: the first step was pre-denaturation at 95°C for 30 s for 1 cycle; the second step was PCR reaction at 95°C for 5 s, 60°C for 15 s, 72°C for 30 s for 40 cycles. Three replicate wells were set up for each sample and the relative expression of the target gene was calculated using the 2-ΔΔCT method. The primers used are as follows: *NUDT10* (forward 5′- CGGTCCGAGAGGTGTACGA -3′, reverse 5′- AATCTTCCCAATCCTCCAGCA -3′).

*METTL1* (forward 5′-GGCAACGTGCTCACTCCAA-3′, reverse 5′- CACAGCCTATGTCTGCAAACT-3′); *NUDT4* (forward 5′-ACCAGTGGATTGTCCCAGGA-3′, reverse 5′-CCCAGAAGTCTGCCTAGTTTTC-3′); *GEMIN5* (forward 5′-CCTCCGTCTTCCTTGTCCG-3′, reverse 5′- CAGAGACCCTTTCGGTGTGTC-3′); *EIF4E1B* (forward 5′-GGACTTCTGGGCGCTATACAG-3′, reverse 5′-CTTGAAGAGGGCGTAGTCACA-3′); and *DCPS* (forward 5′- GCAGCTCCTCAACTAGGCAAG-3′, reverse 5′-GAAGCCGGAGAACGGTAAGC-3′).

## Results

### Identification of Prognostic m7G-Related DEGs and Construction of a Prognostic Model

**Figure 1:**
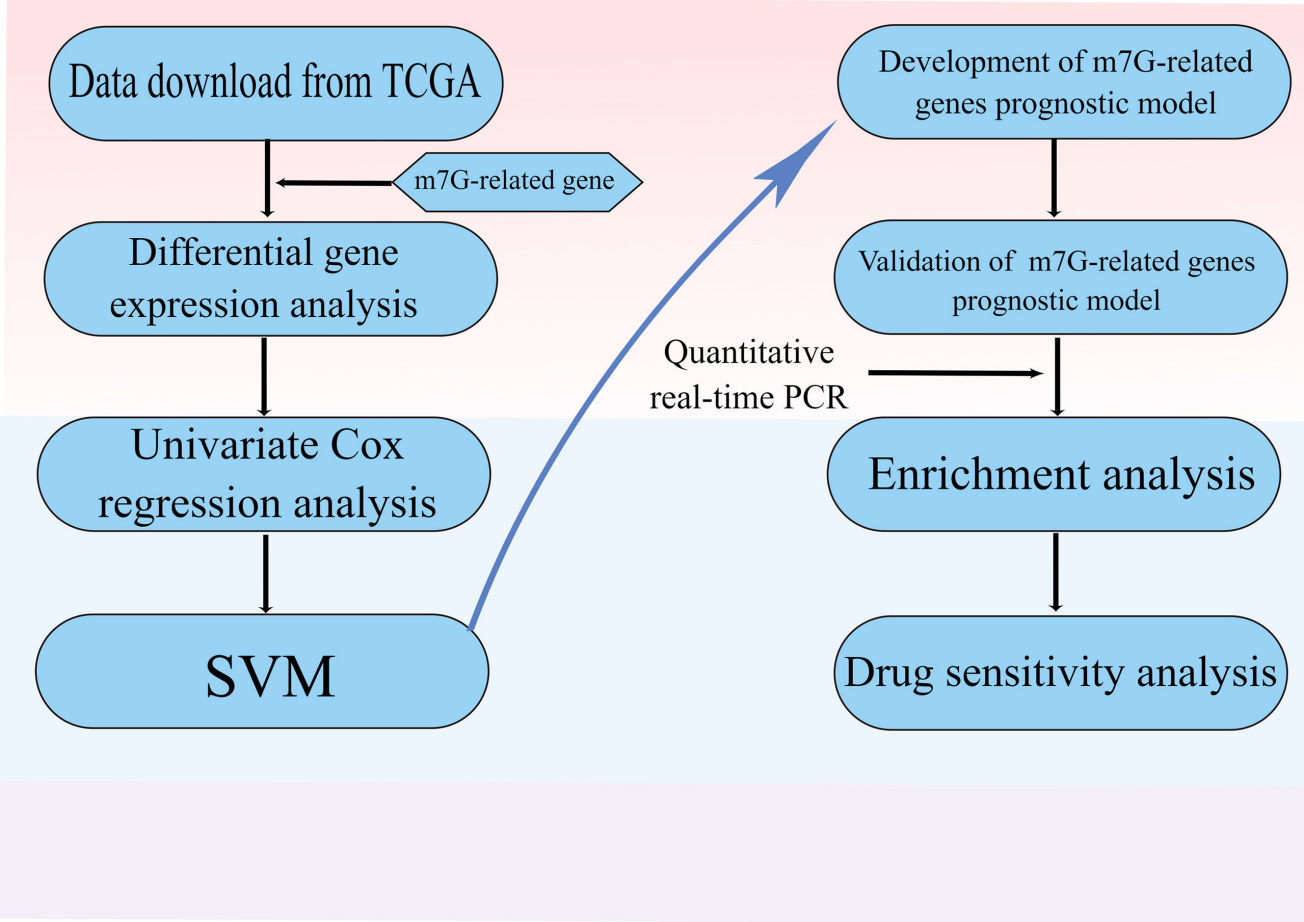
Flow diagram.

**Figure 2:**
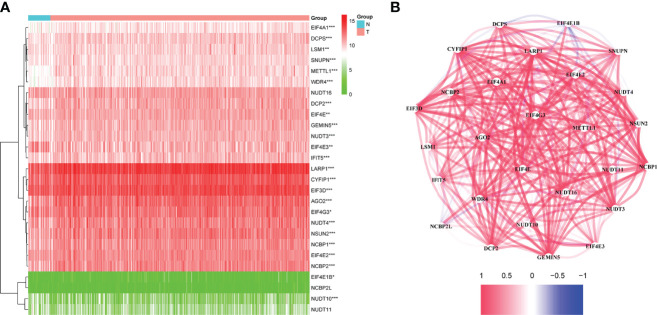
**(A)** Heatmap of the differential m7G-related genes expression in the two groups. **(B)** The relationship between m7G -related DEGs. ns, p > 0.05; *p < 0.05; **P <0.01; ***p < 0.001.

**Figure 3:**
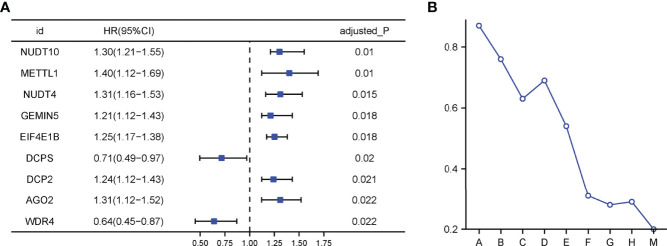
**(A)** Univariate Cox regression analysis. **(B)** Processes of SVM model fitting.

**Figure 4:**
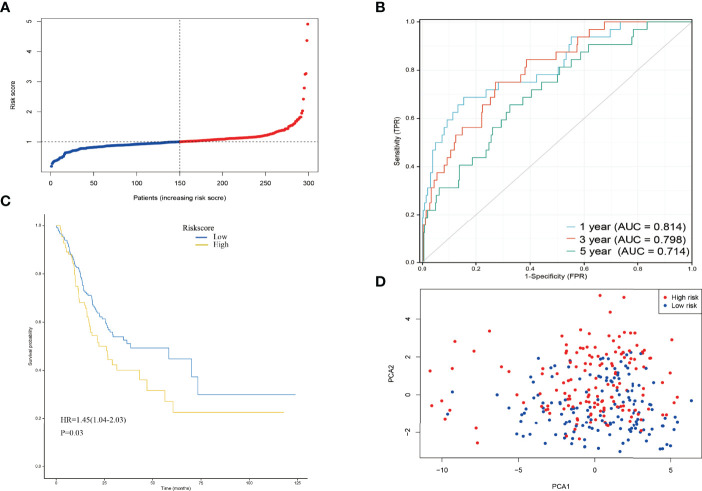
**(A)** The distribution and median value of the risk scores in the derivation cohort. **(B)** AUC of time-dependent ROC curves verified the prognostic performance of the risk score in the derivation cohort. **(C)** Kaplan-Meier curves for the OS of patients in the high-risk group and low-risk group in the derivation cohort. **(D)** PCA plot of the derivation cohort.

The flow chart was shown in **Figure 1**. Thirty-two normal tissue samples and 375 GC tissue samples were obtained from the TCGA database. As per the screening criteria, a total of 20 DEGs were identified out of 29 m7G-related genes (**Figure 2A**, green: low expression level; red: high expression level). **Figure 2B** shows the relationship between different m7G-related DEGs, with a significant positive correlation observed between most m7G-related genes. Nine DEGs were associated with overall survival in the univariate regression analysis(**Figure 3A**). After screening these genes by SVM, six genes were obtained(**Figure 3B**). They were then subjected to a multivariate Cox analysis, weighted according to the relative coefficients in the multivariate Cox regression. The scoring equation was:

Risk score = *NUDT10*×0.021+*METTL1*×0.0223-*NUDT4*×0.00570+*GEMIN5*×1.44+*EIF4E1B*×0.175-*DCPS*×0.0254. GC patients were divided into high-risk and low-risk groups based on the median risk score (**Figure 4A**, median risk score=1.09). In addition, the ROC curve analysis showed that the area under the curve (AUC) was 0.814 at 1 year, 0.798 at 3 years, and 0.714 at 5 years(**Figure 4B**), all of which were greater than 0.7. The calibration curve (**Figure S1**) showed that the prediction results of the prognostic risk score model were in good agreement with the actual observations. The risk model described in this study demonstrates a good degree of discrimination and calibration. The K-M survival analysis showed a significant difference in the survival of the high-risk and low-risk groups (**Figure 4C**, p<0.05), with the high-risk group having a significantly lower survival rate than the low-risk group. The survival rate also decreased over time. PCA analysis showed that the different risk groups were distributed in two directions, suggesting that a six-gene prognostic risk score model could distinguish well between high- and low-risk groups (**Figure 4D**).

### Validation of Prognostic Models

**Figure 5:**
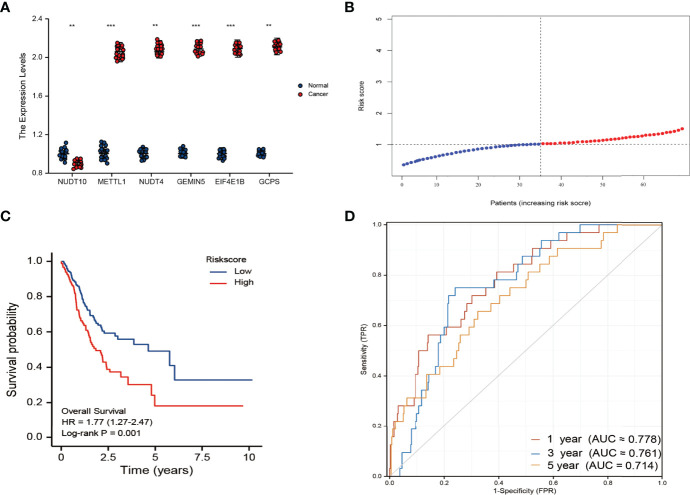
**(A)** Results of qRT-PCR analysis. *p < 0.05; **P <0.01; ***p < 0.001 **(B)** The distribution and median value of the risk scores in the derivation cohort. **(C)** Kaplan-Meier curves for the OS of patients in the high-risk group and low-risk group in the validation cohort. **(D)** AUC of time-dependent ROC curves verified the prognostic performance of the risk score in the validation cohort.

The β’s calculated from the TCGA cohort and the expression levels of m7G-related genes in the validation cohort were used to calculate risk scores for patients in the validation cohort. PCR results demonstrate that six genes are still differentially expressed in the validation cohort(**Figure 5A**). Patients with GC were divided into high-risk and low-risk groups based on the median risk score(**Figure 5B**, median risk score=1.06). The results showed that compared with the low-risk group, the high-risk group had a worse prognosis (**Figure 5C**). Furthermore, the AUC of the 6-gene signature was 0.778, 0.761, and 0.714 at 1, 3, and 5 years, respectively(**Figure 5D**). On a calibration graph, the validation group revealed a moderate calibration (**Figure S2**).

### Independent Prognostic Value of the Prognostic Risk Score Model

Univariate and multivariate Cox regression analyses were performed to evaluate whether the prognostic risk score model could be used as an independent prognostic predictor. The risk score was found to be significantly associated with overall survival (OS) in patients with GC, and can thus be used as an independent prognostic factor for evaluating patients with GC (**Table 3**). Considering the clinical utility of the risk model, we incorporated clinical parameters to establish a nomogram to predict the 1-, 3-, 5- OS (**Figure S3**). The discrimination of the nomogram was assessed using the ROC curve. **Figures S4**, **S5** show the ROC curves of the nomogram in modeling group and validation group respectively, and the results prove that the nomogram have good discrimination.

**Table 3:** Independent prognostic value of the prognostic risk score model. Bold values indicate significant P-values (<0.05)

Characteristics	Univariate analysis		Multivariate analysis	
	Hazard ratio (95% CI)	P value	Hazard ratio (95% CI)	P value
T stage				
T1	Reference			
T2	6.725 (0.913-49.524)	0.061	5.291 (0.711-39.368)	0.104
T3	9.548 (1.326-68.748)	**0.025**	6.219 (0.851-45.464)	0.072
T4	9.634 (1.323-70.151)	**0.025**	5.572 (0.748-41.529)	0.094
N stage				
N0	Reference			
N1	1.629 (1.001-2.649)	**0.049**	1.374 (0.814-2.317)	0.234
N2	1.655 (0.979-2.797)	0.060	1.471 (0.854-2.535)	0.164
N3	2.709 (1.669-4.396)	**<0.001**	2.363 (1.403-3.981)	**0.001**
M stage				
M0	Reference			
M1	2.254 (1.295-3.924)	**0.004**	2.386 (1.310-4.344)	**0.004**
Gender				
Female	Reference			
Male	1.267 (0.891-1.804)	0.188		
Age				
<=65	Reference			
>65	1.620 (1.154-2.276)	**0.005**	1.815 (1.262-2.610)	**0.001**
Riskscore				
Low	Reference			
High	1.764 (1.550-1.961)	**0.048**	1.575 (1.242-1.864)	**0.021**

Bold values indicate significant P-values (<0.05).

### Functional Enrichment Analysis

The differentially expressed m7G-related genes were enriched in 144 biological processes (BP), 16 cellular components (CC), and 30 molecular functions (MF). Among GO-BP, there was significant enrichment of differential genes in the regulation of ‘cellular amide metabolic process,’ ‘regulation of translation,’ and ‘translational initiation.’ Significantly enriched GO-CC included ‘eukaryotic translation initiation factor 4F complex,’ ‘RNA cap binding complex,’ and ‘mRNA cap binding complex.’ The significantly enriched GO-MF included ‘translation initiation factor activity,’ ‘RNA 7−methylguanosine cap binding,’ and ‘RNA cap binding.’ Enrichment analysis by KEGG showed that the differentially expressed genes were mainly enriched in the ‘RNA degradation pathway,’ ‘EGFR tyrosine kinase inhibitor resistance pathway’, and ‘RNA transport pathway’ (**Figure S6**).

### Analysis of Immune Infiltration Patterns in High- and Low-Risk Groups

**Figure 6:**
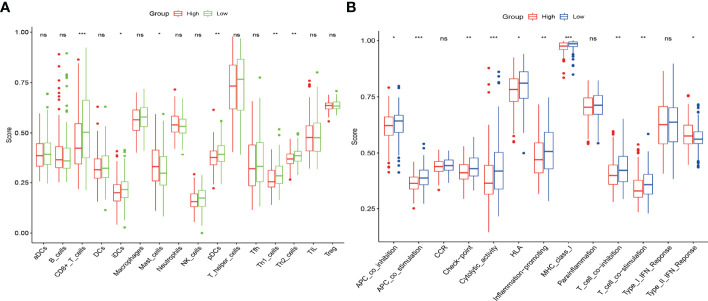
The scores of 16 immune cells **(A)** and 13 immune-related functions **(B)** are displayed in boxplots. *, P < 0.05; **, P < 0.01; ***, P < 0.001; ns, P > 0.05.

**Figure 7:**
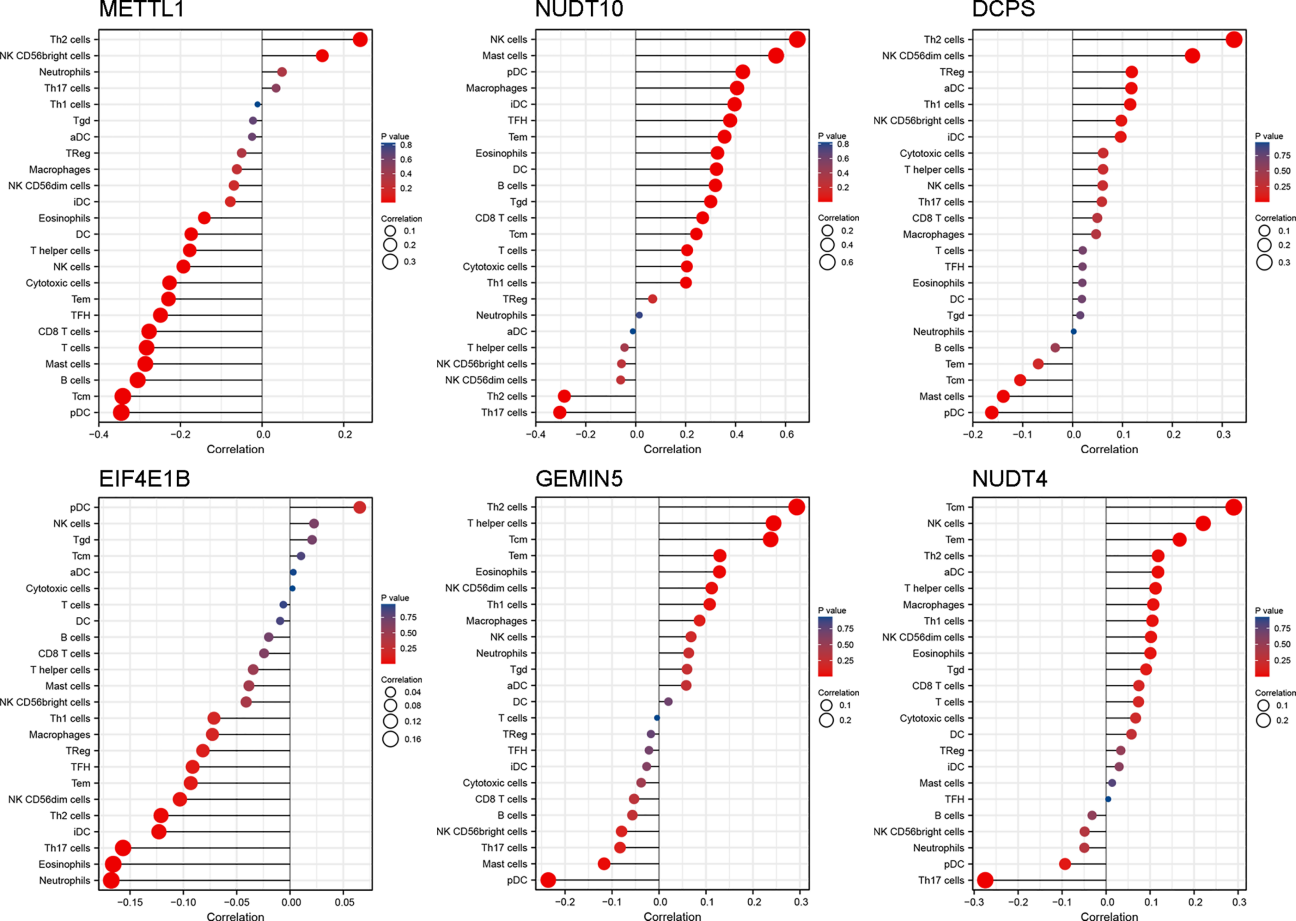
The relationship between genes and immune cells. The color and size of the datapoint represent the direction and significance (P-value) of the correlation.

To further explore the correlation between the prognostic risk score model and immune status, we quantified different immune cell subsets, cell functions, and pathways using ssGSEA. The scores of most immune cells, including CD8+_T_cells, iDCs, Mast_cells, pDCs, Th1_cells, Th2_cells, and macrophages, were significantly different between the high-risk and low-risk groups. Th1_cells, Th2_cells, CD8+ T cells, and APC co-stimulation scores, inflammation−promoting, T_cell_co−stimulation were higher in the low-risk group, indicating that the antigen presentation process and cellular and humoral immunity were better in the low-risk group (**Figures 6A, B**). Therefore, the weakening of anti-tumour immunity in high-risk patients may be a reason for their poor prognosis. We further analysed the relationship of the six genes with immune cells in gastric cancer, and found that *DCPS, GEMIN5*, and *METTL1* had a significant positive correlation with Th2 cells, suggesting that they are synergistic in immune cell activation. In contrast, *EIF4E1B* and *NUDT10* had a negative correlation with Th2 (**Figure 7**).

### Drug Sensitivity Analysis

**Table S1** reveal the candidate drugs from the DSigDB database for the DEGs. Our results suggest that Trichostatin A and scriptaid may serve as a potential agent for the treatment of GC. Trichostatin A and scriptaid are histone deacetylase inhibitors (HDACi) (21). Studies have shown that some HDACi have the ability to resist tumour development through a variety of mechanisms, such as the induction of apoptosis, oxidative stress damage, and autophagy (22–24). Scott showed that HDACi blocked tumour cells in the G1/S phase and inhibited tumour growth (25). Vorinostat inhibits the growth of cancer cells by TGF‐β1 (26).

## Discussion

GC is one of the most lethal solid tumours and is characterised by complex molecular and cellular heterogeneity (27). Currently, the molecular markers related to GC that are widely used in clinical practice include CEA, CA19-9 and CA724 (28); however, the specificity of this tumour marker is low and it does not have the ability to determine prognosis. Therefore, there is an urgent clinical need to develop new biomarkers for GC, which can help in early diagnosis and prognosis, and assist in the formulation of personalised treatment plans. RNA post-transcriptional modifications have lately been the hotspot of research in epigenetics and have received increasing attention in recent years (29). The aim of this study is to construct a prognosis-related gene prediction model for gastric cancer patients by screening survival-related m7G-related genes through high-throughput sequencing combined with bioinformatics.

Among more than 170 RNA post-transcriptional modifications identified so far, two-thirds of them are methylation modifications, including m1A, m6A, m5C, and m7G (29, 30). m7G was originally found in eukaryotic mRNA, tRNA and rRNA (31). As an important regulator of m7G, *METTL1* expression is significantly upregulated in hepatocellular carcinoma and is associated with poor patient prognosis. It is also known to exhibit oncogenic activity through the PTEN/AKT signaling pathway (32). Although several molecular marker-based studies have attempted to predict the prognosis of GC, no study has systematically used m7G-related genes as molecular markers in this patient population. To our knowledge, this is the first study to explore the use of m7G-related genes as molecular markers for predicting the prognosis in patients with GC cancer.

Twenty genes associated with GC prognosis were identified by differential gene expression analysis. Six genes (*NUDT10, METTL1, NUDT4, GEMIN5, EIF4E1B, DCPS*) were finally selected based on univariate and multivariate Cox regression analysis, and used as the basis for constructing a prognostic risk score model. The model was further validated in the training and test sets and found to have relatively high AUC values in predicting patient survival at 1, 3, and 5 years, thereby confirming the good predictive ability of the model. Also, multi-factor Cox regression analysis confirmed that the present model could be used as an independent predictor. The enzyme encoded by the *NUDT10* gene determines the rate of phosphorylation in DNA repair, stress response, and apoptosis (33). *METTL1* exhibits oncogenic activity in hepatocellular carcinoma (24), while in colorectal cancer, it acts as a tumour suppressor (34). In addition, the overexpression of *METTL1* also increased the chemosensitivity of colorectal cancer cells to cisplatin by regulating the miR-149-3p/S100A4/p53 axis (34). These results suggest that maintaining high levels of functional tRNAs may be critical for the role of *METTL1* in cancer cells. *NUDT4* encodes for the enzyme diphosphoinositol polyphosphate phosphohydrolases 2 that controls the turnover of diphosphoinositol polyphosphates, thus regulating intracellular vesicle trafficking and DNA repair (35). *GEMIN5* regulates mRNA splicing and tumour cell motility (36). In addition, *GEMIN5* has a key role in reprogramming cellular translation (37). We selected chemotherapy drugs that are currently used for the treatment of GC evaluated the effect of patients in the two groups to these drugs. A total of nine potential drugs were provided for treating GC.

In recent years, immune infiltration has come into focus as a prognostic indicator. To further explore the correlation between risk score models and immune status, we quantified different immune cell subsets, cell functions, and pathways and concluded that the co-stimulation scores for Th1_cells, Th2_cells, CD8+ T cells, and antigen presenting cells were higher in the low-risk group, indicating that antigen presentation and cellular and humoral immune responses were stronger in this group. Moreover, higher risk scores were associated with impaired anti-tumour immune response. Therefore, diminished anti-tumour immunity in high-risk patients may be a cause of their poor prognosis.

## Conclusions

Based on the correlation between tumour and m7G, six genes were screened for correlation with patient prognosis and used to construct a risk score model, thereby providing new ideas and methods to assess the prognosis of GC.

## Data Availability Statement

The original contributions presented in the study are included in the article/**Supplementary Material**. Further inquiries can be directed to the corresponding author.

## Ethics Statement

All procedures involving human participants were carried out in accordance with the Scientific Research Projects Approval Determination of Independent Ethics Committee of Hospital Affiliated 5 to Nantong University, with the 1964 Helsinki Declaration and its later amendments or comparable ethical standards.

## Author Contributions

X-tY and X-yL carried out experiments, and S-lW, HL, D-hC , J-xY, L-xS, and X-yL wrote the manuscript, X-tY performed manuscript review. All authors contributed to the article and approved the submitted version.

## Funding

This study received Fundamental research program funding of Ninth People’s Hospital affiliated to Shanghai Jiao Tong university School of Medicine (No. JYZZ076), Clinical Research Program of Ninth People’s Hospital, Shanghai Jiao Tong University School of Medicine (No. JYLJ201801, JYLJ201911), the China Postdoctoral Science Foundation (No. 2017M611585) and the National Natural Science Foundation of China (No. 81871458).

## Conflict of Interest

The authors declare that the research was conducted in the absence of any commercial or financial relationships that could be construed as a potential conflict of interest.

The reviewer DC declared a shared affiliation, with no collaboration, with several of the authors, X-yL, L-xS, X-tY, S-lW, HL, J-xY, to the handling editor at the time of review.

## Publisher’s Note

All claims expressed in this article are solely those of the authors and do not necessarily represent those of their affiliated organizations, or those of the publisher, the editors and the reviewers. Any product that may be evaluated in this article, or claim that may be made by its manufacturer, is not guaranteed or endorsed by the publisher.

## Supplementary Material

Supplementary Figure 1 Calibration plots of the prognostic model in the derivation cohort.

Supplementary Figure 2 Calibration plots of the prognostic model in the validation cohort.

Supplementary Figure 3 A Nomogram predicting 1-, 3-, 5-year OS rate.

Supplementary Figure 4 ROC curves of the combined nomogram in the derivation cohort.

Supplementary Figure 5 ROC curves of the combined nomogram in the validation cohort.

Supplementary Figure 6 Analysis of GO and KEGG enrichment for DEGs. The size of bubbles represents the number of genes.
