# Supplementary figures and images for: Construction and Validation of a m7G-Related Gene-Based Prognostic Model for Gastric Cancer
Source: Front Oncol. 2022 Jun 30;12:861412. doi: 10.3389/fonc.2022.861412 (PMC9281447; doi:10.3389/fonc.2022.861412)

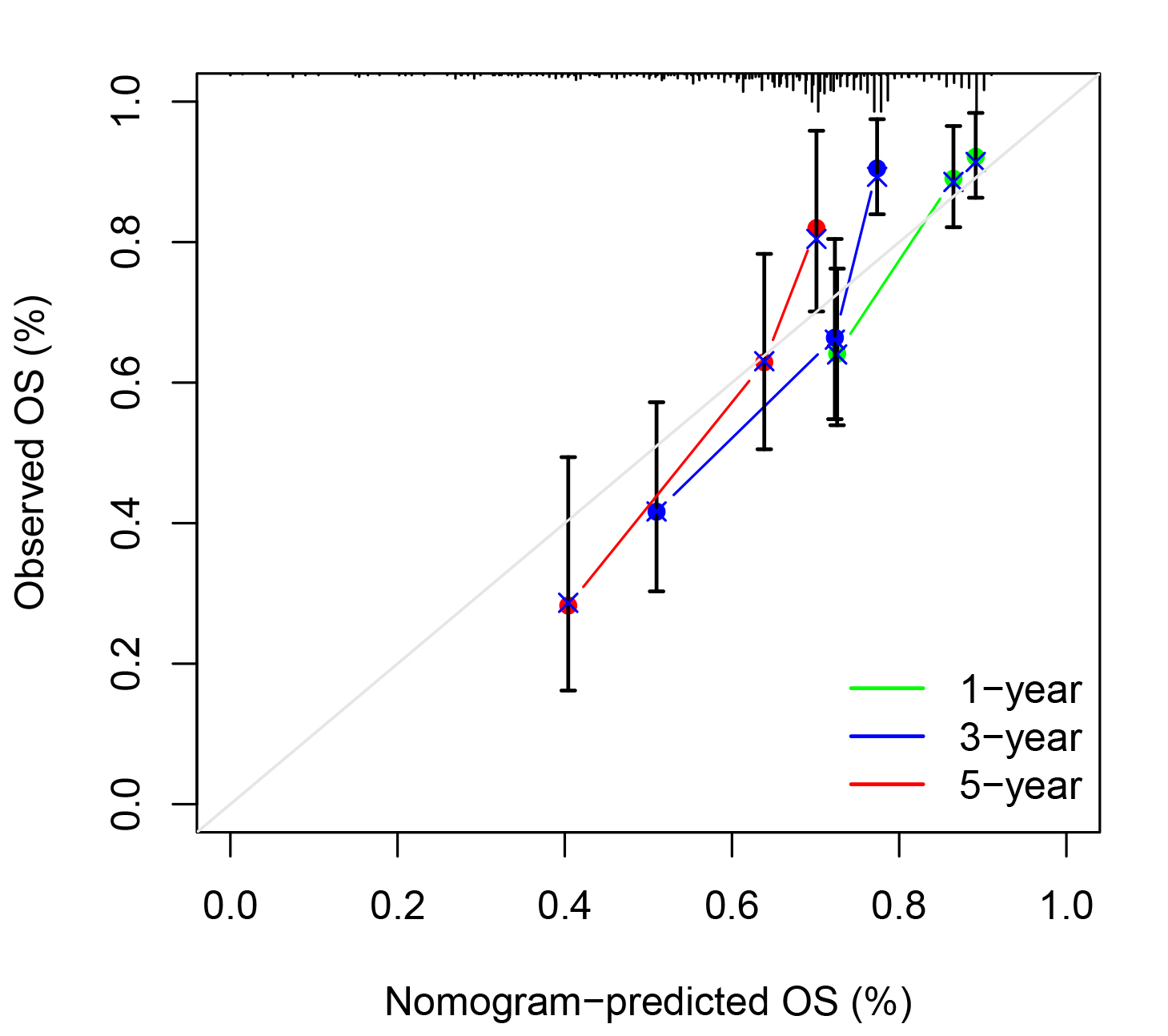

Supplement: Supplementary Figure 1 — Calibration plots of the prognostic model in the derivation cohort. [file Image_1.jpeg]

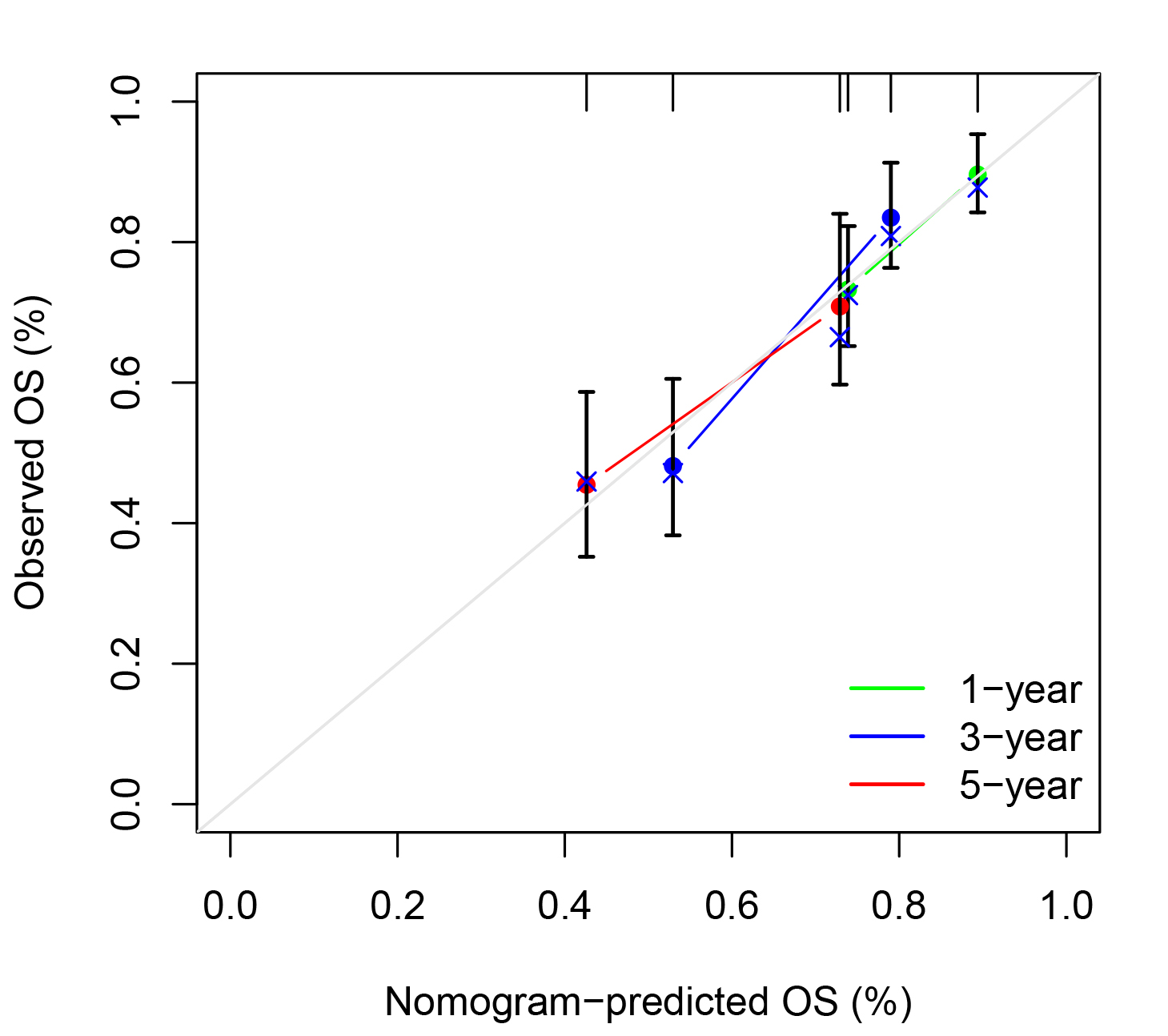

Supplement: Supplementary Figure 2 — Calibration plots of the prognostic model in the validation cohort. [file Image_2.jpeg]

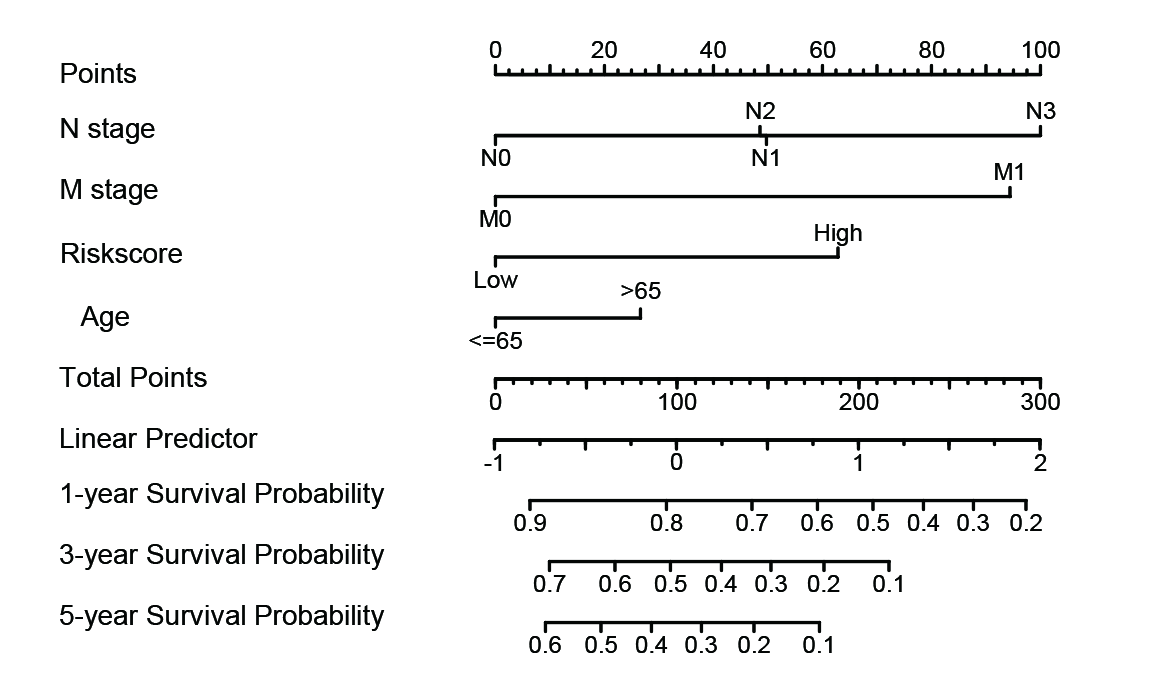

Supplement: Supplementary Figure 3 — A Nomogram predicting 1-, 3-, 5-year OS rate. [file Image_3.tif]

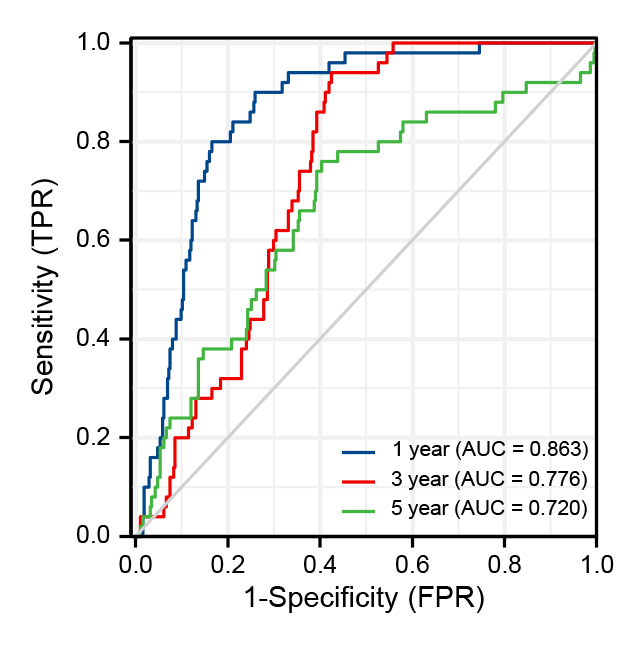

Supplement: Supplementary Figure 4 — ROC curves of the combined nomogram in the derivation cohort. [file Image_4.jpeg]

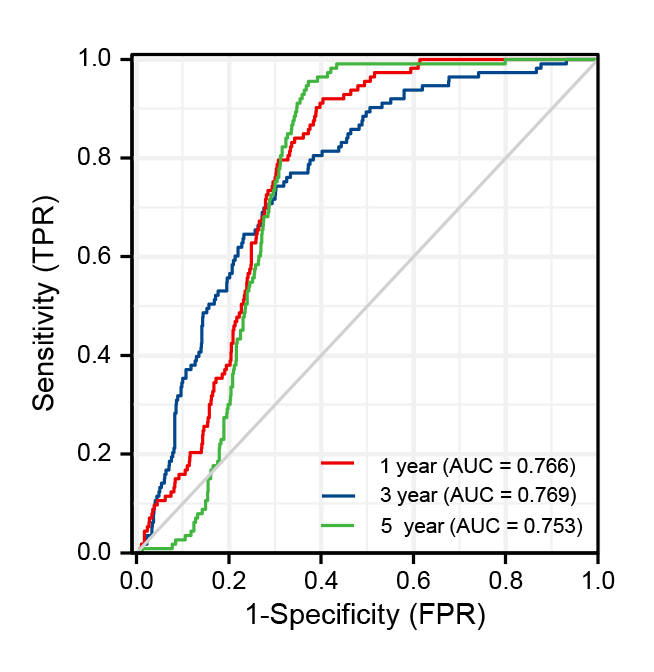

Supplement: Supplementary Figure 5 — ROC curves of the combined nomogram in the validation cohort. [file Image_5.jpeg]

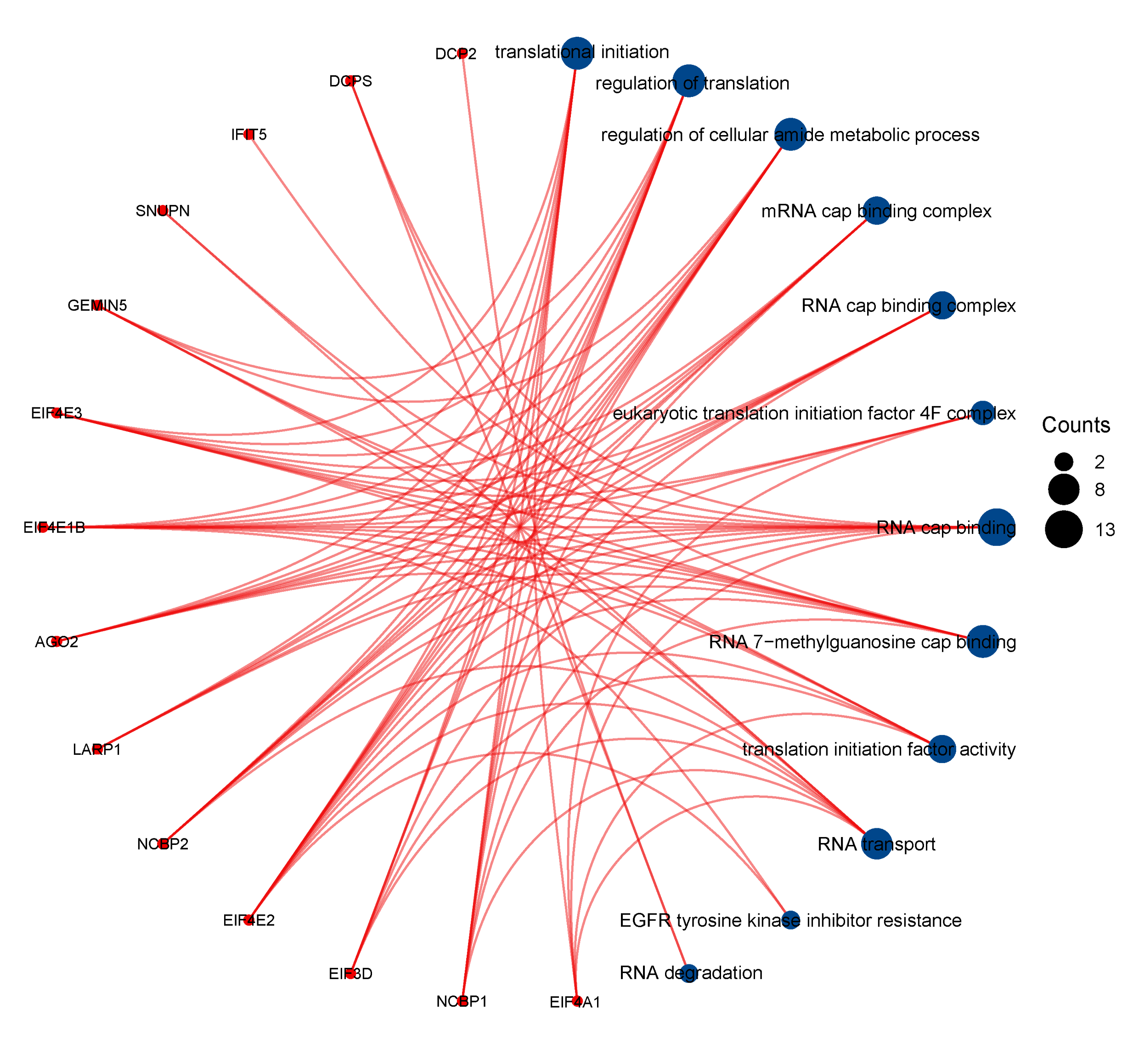

Supplement: Supplementary Figure 6 — Analysis of GO and KEGG enrichment for DEGs. The size of bubbles represents the number of genes. [file Image_6.tif]
