# Supplementary material for: Construction and Validation of a m7G-Related Gene-Based Prognostic Model for Gastric Cancer
Source: Front Oncol. 2022 Jun 30;12:861412. doi: 10.3389/fonc.2022.861412 (PMC9281447; doi:10.3389/fonc.2022.861412)
Supplement: Supplementary file 7 [file Table_1.docx]

**TableS1:** **Drug sensitivity analysis.**

| Term | Adjusted P-value | Combined Score |
| --- | --- | --- |
| rifabutin MCF7 DOWN | 0.001154 | 576.3682 |
| trichostatin A HL60 DOWN | 0.00155 | 177.2873 |
| METHYL METHANESULFONATE CTD 00006307 | 0.00669 | 60.02476 |
| vorinostat HL60 DOWN | 0.014139 | 97.78538 |
| scriptaid MCF7 DOWN | 0.02408 | 75.18726 |
| latamoxef HL60 DOWN | 0.025786 | 47.00882 |
| trichostatin A PC3 DOWN | 0.025786 | 52.7735 |
| ETHYL METHANESULFONATE CTD 00005938 | 0.039338 | 34.75409 |
| glibenclamide HL60 DOWN | 0.047054 | 39.20692 |
